# Genome-wide association study reveals genomic loci of sex differentiation and gonadal development in *Plectropomus leopardus*

**DOI:** 10.3389/fgene.2023.1229242

**Published:** 2023-08-14

**Authors:** Jin Gao, Yongbo Wang, Jinye Liu, Fuxiao Chen, Yilan Guo, Hongji Ke, Xulei Wang, Ming Luo, Shuyuan Fu

**Affiliations:** ^1^ Hainan Academy of Ocean and Fisheries Sciences, Haikou, China; ^2^ Hainan Tropical Ocean University Yazhou Bay Innovation Institute, Sanya, China; ^3^ Hainan Provincial Engineering Research Center for Tropical Sea-Farming, Haikou, China

**Keywords:** *Plectropomus leopardus*, genome-wide association study, sex differentiation, gonadal development, genomic loci, candidate genes

## Abstract

**Introduction:** *Plectropomus leopardus*, a commercially significant marine fish, is primarily found in the Western Pacific regions and along the coast of Southeast Asia. A thorough analysis of the molecular mechanisms involved in sex differentiation is crucial for gaining a comprehensive understanding of gonadal development and improving sex control breeding. However, the relevant fundamental studies of *P. leopardus* are relatively lacking.

**Methods:** In this study, a genome-wide association study (GWAS) was conducted to investigate the genetic basis mechanism of sex differentiation and gonadal developmental traits in *P. leopardus* utilizing about 6,850,000 high-quality single-nucleotide polymorphisms (SNPs) derived from 168 individuals (including 126 females and 42 males) by the genome-wide efficient mixed-model association (GEMMA) algorithm.

**Results:** The results of these single-trait GWASs showed that 46 SNP loci (-log_10_ *p* > 7) significantly associated with sex differentiation, and gonadal development traits were distributed in multiple different chromosomes, which suggested the analyzed traits were all complex traits under multi-locus control. A total of 1,838 potential candidate genes were obtained by considering a less-stringent threshold (-log_10_ *p* > 6) and ±100 kb regions surrounding the significant genomic loci. Moreover, 31 candidate genes were identified through a comprehensive analysis of significant GWAS peaks, gene ontology (GO) annotations, and Kyoto Encyclopedia of Genes and Genomes (KEGG) pathway analyses, including *taf7*, *ddx6*, *apoeb*, *sgk1*, *a2m*, *usf1*, *hsd3b7*, *dll4*, *xbp1*, *tet3*, *esr1*, and *gli3*. These trait-associated genes have been shown to be involved in germline development, male sex differentiation, gonad morphogenesis, hormone receptor binding, oocyte development, male gonad development, steroidogenesis, estrogen-synthetic pathway, etc.

**Discussion:** In the present study, multiple genomic loci of *P. leopardus* associated with sex differentiation and gonadal development traits were identified for the first time by using GWAS, providing a valuable resource for further research on the molecular genetic mechanism and sex control in *P. leopardus*. Our results also can contribute to understanding the genetic basis of the sex differentiation mechanism and gonadal development process in grouper fish.

## 1 Introduction

Sex differentiation in aquatic fish is complex and elusive due to the interactive influence of heredity and environment. In teleost, a vast range of reproductive systems gives rise to an enormous plasticity of sex differentiation and change (Devlin and Nagahama, 2002). Elucidating the sex differentiation mechanisms and gonadal transformation into either a testis or an ovary in fish is important for both theoretical study and practical production (Xu et al., 2021). Various prominent genetic and environmental factors are involved during sex differentiation. At the genomic level, some candidate genes have been characterized as well-known factors and major determinants in sex differentiation. On the other hand, multiple physical and chemical environmental factors, such as water temperature, PH, salinity, and social interaction, have been demonstrated to influence sex (Baroiller et al., 2009; Rajendiran et al., 2021). Significantly, the fundamental and molecular mechanisms of sex differentiation in fish show high variability (Piferrer and Guiguen, 2008). To a certain extent, sex differentiation is usually visualized by gonadal differentiation that can take many forms in fish (Devlin and Nagahama, 2002); for example, some gonochoristic species develop only testis or ovaries and remain the same sex throughout their lifespans, some hermaphroditic species possess both sexes of immature gametes in a single gonad or functional sex reversal, and some species show various patterns and an extremely flexible process of sex differentiation. Revealing the molecular mechanisms of sex differentiation in fish will have important implications for research on reproductive development, sex-ratio control, and selective breeding. The gonad development process is an important manifestation of sex differentiation, which requires the involvement of genes and hormones (Mustapha et al., 2021). Accordingly, to comprehensively understand the mechanisms of sex differentiation (SDIF), some indicators of gonadal development, such as gonadal weight (GW) and gonadosomatic index (GSI), are usually considered together for relevant studies of sex-related traits in fish (Wang et al., 2021).

Many research ideas and basic methods with different formats have been used to solve the scientific problem and explore the fundamental mechanisms involved in the genetic information of sex-related traits in aquatic animals. Sex differentiation is a very important part of sex-related traits, which drives the primitive gonad to the testis or ovary (Stévant and Nef, 2019). Histological observation has been a conventional research methodology employed to investigate sex differentiation in gonochoristic fishes (Takashima et al., 1980). Using histological studies to inspect the different stages involved in the development of gonads can provide more detail about the morphological process of sex differentiation (Patil and Nayak, 2021). Moreover, model fish with a deficiency of an important sex-related gene were used to elucidate genetic mechanisms in the development of sexual traits (Zhai et al., 2018). With the development of molecular biology and high throughput sequencing technology, the study of sex differentiation has entered the era of omics. RNA-Seq has undoubtedly become the most popular and inexpensive option for operating omics data analysis. By using gonad transcriptome analysis of male and female channel catfish gonads during the critical period of sex differentiation, a few candidate genes and potential pathways underlying male-preferential cell development were revealed in catfish (Zeng et al., 2016). To uncover the genetic framework underlying sex differentiation of tilapia, a dynamic co-expression network analysis with RNA-Seq was carried out to detect differently expressed genes that were involved in sex differentiation (Tao et al., 2018). A high-quality gonadal transcriptome analysis of Siberian sturgeon was used to search for sex-differentiation genes at undifferentiated stages, and multiple genes related to the stem-cell niche and sex-specific nerve development were screened out (Vizziano-Cantonnet et al., 2018). However, we can only estimate the expression level for each gene with RNA-Seq without regard for the exact relationship between genes and observable characteristics of each individual across the population.

Genetic studies based on genome-wide scans have provided a new approach to sex differentiation in fish. Whole-genome wide association study (GWAS), which identifies associations of genotypes with phenotypes by using statistical inference for differences in the allele frequency of candidate genomic loci among objective populations (Uffelmann et al., 2021), is the most popular method of whole-genome scanning. Compared to previous linkage-based genome-wide studies, the GWAS approach is more powerful and sensitive for locating genomic loci associated with phenotypes (Talukder et al., 2019). To date, GWASs have been widely used to detect genetic loci or genes of sex-related traits in fish. For instance, several sex-related candidate genes within a sex-associated region were identified in *Takifugu bimaculatus* using GWAS (Patil and Nayak, 2021). An approximate GWAS result by ddRAD sequencing has also been obtained in a random breeding population of Larimichthys crocea, some candidate genes (i.e., *dmrt1*, *dmrt3*, *piwil2*, *fam102a*, and *odf2*) and some others (i.e., *axl*, *cyp2a10*, and *cyp2g1*) were detected separately to be involved in sex determination and GSI traits (Lin et al., 2021). Furthermore, a GWAS was conducted to reveal novel sex-related candidate genes (i.e., *trap1* and *fryl*) involved in sex determination and differentiation in *Mesocentrotus nudus* using the genotyping-by-sequencing method (Wang et al., 2022). In addition, similar studies have been carried out on *Cynoglossus semilaevis* (Zhang et al., 2020), *Salmo salar* (Gutierrez et al., 2015; Gabián et al., 2019), and *Scophthalmus maximus* (Martínez et al., 2021). All these GWAS results provide potential genomic candidate loci and genes for dissecting molecular genetic mechanisms of sex-related traits in fish; they could be used to improve reproductive strategy and breeding efficiency.

The leopard coral grouper, *Plectropomus leopardus*, is a high-value species with bright external coloration that belongs to the Serranidae family. According to the available information, *P. leopardus*, the characteristic group of grouper species, exhibits a model of sex differentiation that undergoes a transition from ovary to intersexual gonad and then to testis (Zhou and Gui, 2010). Earlier studies have shown that the sex ratio, sex-specific size, and age structure of *P. leopardus* are variable with geographical location (Adams et al., 2000). However, the mechanism governing sex differentiation and gonadal development of *P. leopardus* is still poorly understood. Therefore, it is of interest to study the underlying genetic architecture of the sex differentiation mechanism in *P. leopardus*. Here, we performed GWAS of sex differentiation and gonadal development traits (GW and GSI) to identify relevant candidate genomic loci and genes.

## 2 Materials and methods

### 2.1 Experimental fish and library construction

All individuals of *P. leopardus* used in this work were obtained from the Hainan Ocean and Fisheries Sciences Research Base in Qionghai City, Hainan Province, P.R. China. The study population was the 4-year-old *P. leopardus*, which is the second filial generation of a breeding population collected from different regions throughout Hainan Island in 2009. A total of 168 individuals were sampled from the population for genomic analysis. Genomic DNA of each sample was extracted and purified from caudal fin samples using the standard phenol–chloroform protocol (Sambrook and Russell, 2006). Then, the quality of isolated genomic DNA was verified by 1% agarose gel electrophoresis and a Qubit™ dsDNA HS Assay Kit (Life Technologies, CA, United States of America). The sequencing libraries were constructed using a Nano DNA HT Sample Prep Kit (Illumina United States of America) with a total amount of 0.3 μg DNA per sample.

### 2.2 Whole genome resequencing and SNP calling

The fragments with 350 bp in size of genomic DNA samples were isolated by sonication, then end-polished, A-tailed, and ligated with the full-length adaptor for Illumina sequencing with further PCR amplification. Pair-end sequencing was performed to generate raw sequencing reads, followed by quality control using the fastp v0.20.1 (Chen et al., 2018). Then, the GATK v4.1.5.0 (Mckenna et al., 2010) was applied to split the pooling sequencing data and genotyping. For SNP calling, the sequencing reads were aligned to the *P. leopardus* genome (CNGBdb Accession No. CNP0000859), and SNPs were identified by HaplotypeCaller of the software package GATK. We used QC-Chain to filter the raw reads by discarding the adapter sequences, low-quality reads, and duplicated reads, and then all indels and non-biallelic SNPs were removed.

### 2.3 Statistics of phenotype data

In this study, 168 experimental fish were euthanized to measure body weight (BWE), after which they were dissected to determine gonadal weight (GW) and assess physiological sex. We used the GSI to indicate sexual maturity, which represented the gonadal development of individuals, by the formula below (Lin et al., 2021):

$$GSI=\frac{Gonadal\;Weight\left(GW\right)}{Body\;Weight\left(BWE\right)}\times 100.$$

To distinctly perform a GWAS of GW and GSI for investigating the gonadal development process and to avoid sex differences affecting the results of the association analysis, a statistical analysis of male and female phenotypes, including female gonadal weight (FGW), male gonadal weight (MGW), female gonadosomatic index (FGSI), and male gonadosomatic index (MGSI), was conducted. Wilcox tests were implemented in GW and GSI between female and male individuals by R to ensure there were significant differences between the physiological sexes.

### 2.4 Statistical model

GWASs were conducted for each of the SDIF, GW, GSI, FGW, MGW, FGSI, and MGSI traits by univariate linear models (ULMs) using a genome-wide efficient mixed model association (GEMMA) algorithm. For the phenotypic traits influenced by gender (GW and GSI), sex difference (female or male) was considered as a fixed covariate or not in the statistical methods. Each phenotype could be explained by the following linear mixed model:

y=Wα+xβ+Zu+ε,

where **y** is the *n* × 1 vector of phenotypic values of each trait, *n* is the number of individuals, **W** is the *n* × *c* matrix of covariates (fixed effects) such as a column vector of 1 and sex difference for GW and GSI, **α** is the *c* × 1 vector of corresponding coefficients of fixed factors, *c* is the number of covariates, **x** is the *n* × 1 vector of SNP marker genotypes, *β* is the effect size of the marker, **Z** is an *n*×*m* incidence matrix, **u** is an *m* × 1 vector of random polygenic effects while excluding the tested SNP, and **ε** is the *n* × 1 vector of random residual errors. Manhattan and quantile–quantile (QQ) plots were used to visualize potential regions of interest that are associated with the phenotype and assess whether or not the dataset matches a specified probability distribution. All Manhattan and QQ plots in this study were drawn using R v4.0.5.

### 2.5 Potential candidate genomic loci and gene searching

To identify potential candidate genomic loci significantly associated with sex differentiation and gonadal development traits, the threshold *p*-value (-log_10_ *p* > 7) was used to calculate the genome-wide significance of each SNP marker. After the candidate genomic loci were screened out, we searched candidate genes based on the 100-kb regions surrounding the most significant SNPs according to their locations and functions. The less-stringent threshold *p*-value (-log_10_ *p* > 6) was also used for significant SNP identification. Subsequently, the candidate genes inferred from these SNPs were applied to explore the potential association with each objective trait by GO function annotation and KEGG pathway enrichment. All SNPs associated with the candidate genes involved in the aforementioned GO term were selected to compare with GWAS peaks on regions (±100 kb) with multiple SNPs exceeding the threshold in each trait. Finally, coincident SNPs were screened out as the most valuable genomic loci that were explicitly associated with sex differentiation and gonad development.

## 3 Results

### 3.1 Sequencing data and genotyping

Genomic DNA of 168 *P. leopardus* samples were sequenced by Illumina NovaSeq 6000 platform (Illumina, CA, United States) to generate 4.35 billion raw reads (∼1304.04 Gb in length). After the data filtering was conducted, 4.28 billion clean reads (∼1284.05 Gb in length) were obtained. A total of 14,653,768 SNPs of 168 samples were identified after filtering by VCFtools v0.1.15 (Danecek et al., 2011). Moreover, SNPs were further filtered by PLINK1.90 v1.90b4 (Purcell et al., 2007) with the parameters of “--mind 0.15 || --geno 0.10 || --maf 0.05 || --hwe 0.0001.” In addition, we used Beagle v5.4 (Browning et al., 2021) to impute all missing SNPs with default parameters. Finally, 168 samples with more than six million SNPs (6,890,254 SNPs for SDIF, GW, and GSI; 6,823,720 SNPs for FGW and FGSI; and 6,431,681 SNPs for MGW and MGSI) remained for the GWAS research.

### 3.2 Statistics of phenotypic analysis

**FIGURE 1:**
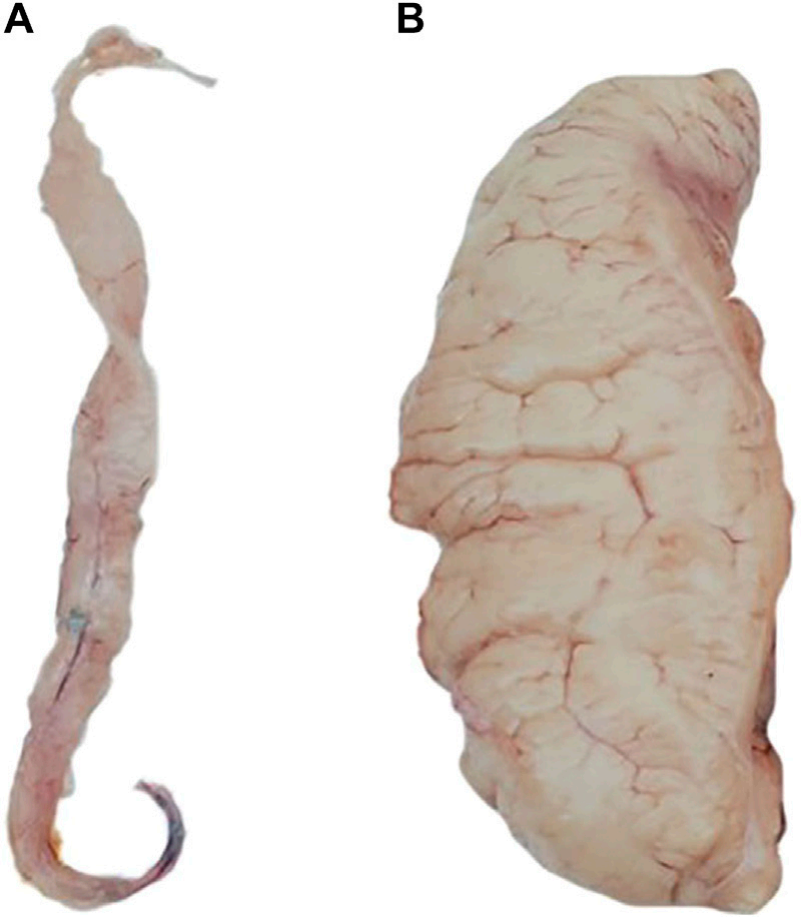
**(A)** Male gonad and **(B)** female gonad of *P. leopardus*.

The phenotypic records of 168 individuals for each trait were counted in Table 1. According to the statistical analysis, we found a significant difference between the phenotype of FGW, which is 131.71 ± 85.91 g, and the phenotype of MGW, which is 10.10 ± 5.05 g. The visual difference between male and female gonads is shown in Figure 1. A significant difference was also presented between the phenotype of FGSI (5.04% ± 2.75%) and MGSI (0.41% ± 0.23%). By using the Wilcoxon test, the significant differences (*p* < 0.0001) were proved between the female and male groups of the GW and GSI phenotypes (Supplementary Figure S1). The coefficient of variation (CV) for each phenotypic trait value was greater than or equal to 0.5, which indicated that all traits possessed relatively discrete phenotypes. However, gender-specific statistics for GW and GSI might help decrease the CV of the phenotypes (Table 1).

**TABLE 1:** Statistical details of the related quantitative traits of sex differentiation.

Trait	N	Max	Min	Mean ± SD	SE	CV
GW (g)	168	382.00	2.00	101.30 **±** 91.21	7.03	0.90
GSI (%)	168	11.89	0.08	3.89 **±** 3.12	0.24	0.80
FGW (g)	126	382.00	20.00	131.71 **±** 85.91	7.65	0.65
MGW (g)	42	26.00	2.00	10.10 **±** 5.05	0.78	0.50
FGSI (%)	126	11.89	0.73	5.04 **±** 2.75	0.24	0.54
MGSI (%)	42	1.47	0.08	0.41 **±** 0.23	0.04	0.56

Note: N is the number of samples, Min is minimum, Max is maximum, SD is standard deviation, SE is standard error, and CV is coefficient of variation.

### 3.3 Genome-wide association study

**FIGURE 2:**
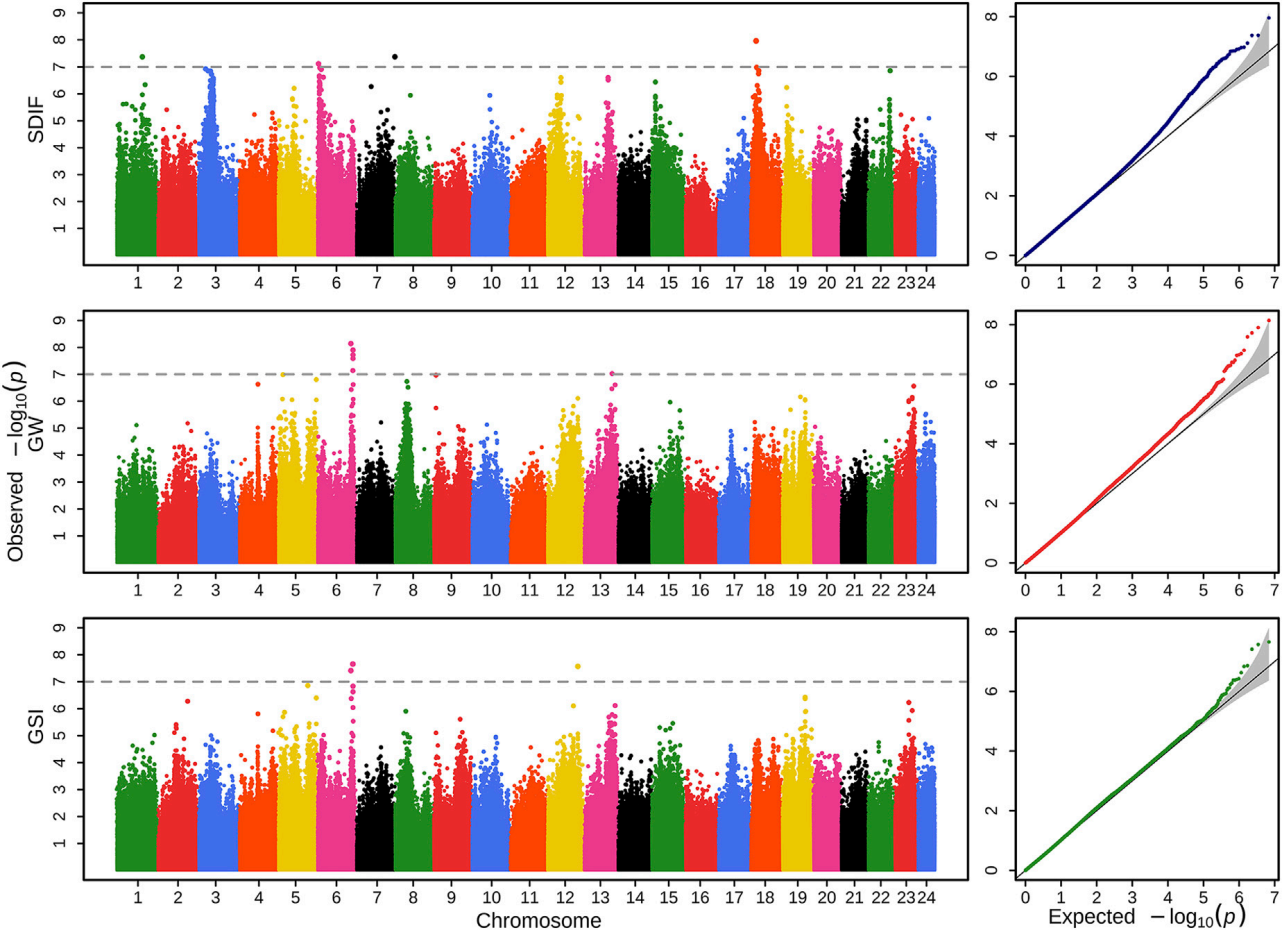
Manhattan and QQ plots of genome-wide SNP significance for the traits of SDIF, GW, and GSI in *P. leopardus*. The *X*-axis shows all SNPs across chromosomes 1–24, and the *Y*-axis represents the -log_10_ *p*. The gray dotted line indicates the genome-wide significant thresholds (*p* < 10E-7).

To investigate the genomic loci and genes significantly associated with sex differentiation and gonadal development process, GWAS on SDIF and a total of eight quantitative traits, including GW, GSI, FGW, MGW, FGSI, and MGSI, were conducted by using a linear mixed-model. Furthermore, in order to clarify the impact of gender on GW and GSI, the physiological sex of each individual was taken as the covariable of the GWAS model. Therefore, GW and GSI with physiological sex as covariates (GW-CoSex and GSI-CoSex) were added to the analysis. Manhattan and quantile–quantile (QQ) plots of nine specific traits are shown in Figure 2 and Supplementary Figure S2. In addition, genomic control (GC) values of each analysis were estimated to evaluate false negatives or false positives in GWAS (Supplementary Table S1). Based on the genome-wide threshold of -log_10_ *p* > 7, we identified 46 SNPs that significantly correlated with sex differentiation and gonadal development traits (Supplementary Table S2). Here, four SNPs associated with the SDIF trait were located on chromosomes 1, 6, 7, and 18 (Figure 2). For GW and GSI traits, six (located on chromosomes 6 and 13) and three (located on chromosomes 6 and 12) SNPs showed a significant correlation, respectively (Figure 2). To consider the effect of physiological sex on gonadal development, GWASs of GW-CoSex and GSI-CoSex were implemented to identify five (located on chromosomes 5, 8, and 13) and eight (located on chromosomes 2, 5, 6, and 13) significant SNPs (Supplementary Figure S2). To avoid sex differentiation affecting the associated results of GW and GSI, we also separately conducted the statistical analysis of male and female phenotypes, whereafter four significantly associated SNPs that exceeded the threshold were found in FGW (located on chromosome 13), two were found in MGW (located on chromosome 7), seven were found in FGSI (located on chromosomes 1, 2, 5, and 13), and 17 were found in MGSI (located on chromosomes 1, 2, 6, 9, 12, 13, 15, 16, 17, and 21) traits (Supplementary Figure S2).

### 3.4 Identification and functional characterization of genomic loci and candidate genes

**FIGURE 3:**
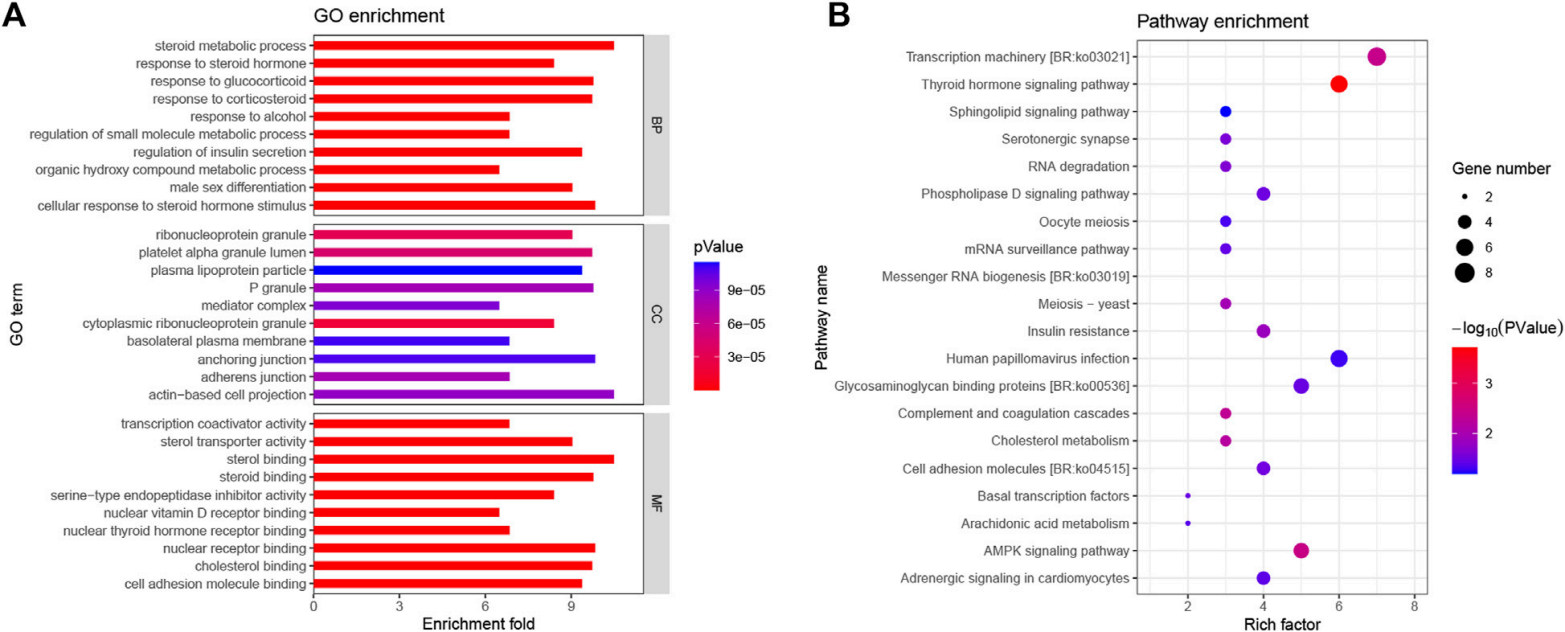
GO and KEGG analysis of the 156 potential candidate genes associated with sex differentiation and gonad development of *P. leopardus*. **(A)** Top 10 most significant GO terms (BP, biological process; CC, cellular component; and MF, molecular function) associated with the candidate genes; **(B)** top 20 most significant KEGG pathways associated with the candidate genes.

To conduct a less-stringent threshold of -log_10_ *p* > 6 and ±100 kb regions surrounding the significantly associated loci, we obtained 595 potential candidate genes for SDIF, GW, and GSI traits that play an important role in illuminating the molecular mechanisms of SDIF and 1,243 potential candidate genes for GW-CoSex, GSI-CoSex, FGW, MGW, FGSI, and MGSI that have an effect on the gonadal development process. Subsequently, gene ontology (GO) analyses of the two groups of candidate genes were applied to excavate the functional association with the objective traits. In detail, 88 GO terms (SDIF, GW, and GSI) and 143 GO terms (GW-CoSex, GSI-CoSex, FGW, MGW, FGSI, and MGSI) were annotated as related to sex differentiation and gonad development, such as female/male sex differentiation, female/male gonad development, gonad morphogenesis, steroid biosynthetic process, hormone biosynthetic process, oocyte construction, spermatid differentiation, etc., whereafter 156 candidate genes probably associated with multiple biological processes of sex differentiation and gonad development were selected for GO and KEGG signaling pathway (KEGG) analyses (Figure 3). Accordingly, 23 coincident SNPs have been identified as the most valuable genomic loci from 156 candidate genes with related functional characterization, and GWAS peaks on regions (±100 kb) with at least two SNPs exceeding the threshold (-log_10_ *p* > 6) (Table 2).

**TABLE 2:** Selected significant candidate genomic loci associated with the nine analyzed traits.

Trait	Chr	Position (bp)	Major allele	Minor allele	-log_10_ *p*	Gene
SDIF	3	12179884	A	G	6.27	*cops6*, *itga6*
	3	15479595	A	G	6.58	*arhg6*, *taf7*
	6	2168021	T	C	6.67	*apbp2*, *brip1*
	12	14467040	T	C	6.60	*ddx6*, *apoeb*
	13	25726958	G	A	6.60	*sgk1*
GW	5	40241714	T	C	6.80	*ppm1e*
	6	35489335	G	C	8.14	*a2m*, *usf1*
	6	37752513	C	T	7.90	*mtnr1b*, *akap1*
	19	24116867	G	C	6.06	*hsd3b7*
GSI	6	37752485	A	T	7.74	*mtnr1b*, *akap1*
	19	24113876	C	T	6.64	*hsd3b7*
GW-CoSex	5	40241714	T	C	6.91	*ppm1e*
	6	37752513	C	T	7.86	*mtnr1b*, *akap1*
	13	28067114	C	G	9.22	*dll4*
	13	28849190	T	A	8.16	*lce*
GSI-CoSex	5	6587613	T	A	6.13	*xbp1*
	5	32767410	C	G	7.37	*tet3*, *npffr2*
	5	40241714	T	C	6.83	*ppm1e*
	13	14147226	A	G	6.42	*exoc8*, *arv1*, *sprtn*
	13	28849190	T	A	6.05	*lce*
FGW	-	-	-	-	-	-
MGW	-	-	-	-	-	-
FGSI	5	32767357	T	C	7.39	*tet3*, *npffr2*
	13	28067114	C	G	8.03	*dll4*
MGSI	10	36239938	A	G	6.08	*cetp*
	10	39094333	G	A	7.06	*mkb*, *sall1*
	12	10160189	C	G	9.40	*cnot3*, *sphk2*
	13	20443832	T	A	7.25	*esr1*
	21	19871070	T	C	6.19	*oprk1*
	21	21198422	A	G	7.05	*gli3*

Note: Gene contains the abbreviations of gene names; the complete gene annotations of each gene name are shown in Supplementary Table S3.

## 4 Discussion

The formation mechanism of sex differentiation and transition of *P. leopardus* is a complex subject. By examining the gonads of physio-sexual dimorphic sex-changing individuals, *P. leopardus* first differentiated as an immature female, and then a male-type gonad would be derived from a female-type mature gonad through sex change surgery (Adams, 2003). Early sex change is considered a latent defect for *P. leopardus*, as it shortens the female reproductive period while affecting the male sperm quality. Late sex change, on the other hand, is an untapped potential economic trait in *P. leopardus* because it would bring about higher female fecundity and efficient utilization of energy for reproduction. The sex determination, sex differentiation, or gonadal development mechanisms have been broadly applied in the explanation of the genetic mechanism of aquatic animals by using GWAS. It is interesting to note that existing GWASs on sex-related traits of fish were implemented for various phenotypes, such as sex determination, gonadal development, and sex reversal. Several sex-associated genomic regions and genetic loci related to the sex-determination system and putative sex-determination genes were detected in *Protosalanx hyalocranius* (Li et al., 2020) and *Thunnus orientalis* (Nakamura et al., 2021). Moreover, a single locus with a large controlling effect was identified through GWAS to elucidate the mechanism of sex reversal in *Cynoglossus semilaevis* (Jiang and Li, 2017). Gonadal development is a significant feature when elaborating sex differentiation, which is triggered by sex-determination signals. Gonadal weight and gonadosomatic index were representative phenotypes for identifying genomic loci of sex-related traits in fish (Gao et al., 2018; Lin et al., 2021; Patil and Nayak, 2021). The male heterogametic sex-determination system as a special sex-linked inheritance was also interpreted using GWAS in *Oncorhynchus mykiss* (Fraslin et al., 2020) and *Clarias macrocephalus* (Nguyen et al., 2021).

*Plectropomus leopardus*, a grouper from the subfamily Epinephelinae, has commanded high market prices locally and overseas. Since the rapid increase in market demand, its culture area continues to grow, and breeding technology continues to improve. However, knowledge of the sex differentiation mechanism of this species is still unclear. Therefore, to investigate the molecular mechanisms of sex differentiation and gonadal development traits of *P. leopardus*, we performed GWASs using 168 sexually mature individuals (126 females and 42 males) and more than six million sequencing-based SNPs in this study. The traits to be analyzed were divided into three categories, such as sex differentiation, gonadal weight, and gonadosomatic index, for thoroughly exploring molecular genetic mechanisms of sex development in *P. leopardus*. Especially, phenotypic sex was considered as a covariate, and sex-specific phenotypes were separately conducted for the GW and GSI traits to avoid sex differences affecting the results of association analysis.

In order to identify more genomic loci associated with the analyzed traits, the stringent threshold *p*-value (-log_10_ *p* > 7) and less-stringent threshold *p*-value (-log_10_ *p* > 6) were used for screening significant loci. Afterward, 46 significant SNPs exceeded the stringent threshold, and 250 significant SNPs were positioned above the less-stringent threshold. Moreover, we identified 156 candidate genes within or very close (±100 kb) to the 250 significant SNPs. GO and KEGG enrichment analysis of these candidate genes suggested that GO terms and pathways related to steroid and thyroid hormones were important for sex differentiation and gonadal development. Furthermore, 23 intersecting loci and 31 nearby genes on multiple chromosomes were extracted from functional enrichment results and GWAS peaks. These results indicated that sex differentiation and gonadal development of *P. leopardus* are complex traits under multi-locus control.

For the GWAS of the SDIF trait in this study, we identified nine trait-associated genes that were mainly involved in such germline development, male sex differentiation, gonad morphogenesis, hormone receptor binding, oocyte development, male gonad development, the steroid metabolic process, and the response to glucocorticoid. *itga6* (integrin alpha 6) has been identified as a surface marker of stem spermatogonia for fertility preservation, and its expression in the male germinal epithelium has been confirmed (Sá et al., 2013). It has also been proved that *taf7l*, as a paralogue of *taf7* (transcription initiation factor TFIID subunit 7), cooperates with *trf2* to regulate spermiogenesis (Zhou et al., 2013), and the variation of *taf7* affects the genetic etiology of idiopathic male infertility (Pal et al., 2021). As reported, *ddx6* (probable ATP-dependent RNA helicase DDX6) belongs to a family of DEAD-box RNA helicases that was highly expressed during various stages of gonad development in the Chinese mitten crab (*Eriocheir sinensis*) (Li et al., 2015). Based on the ontogenic gonadal transcriptomes, the mechanisms of sex change of the ricefield eel (*Monopterus albus*) have been elucidated (Fan et al., 2022), and the expression of *apoeb* (apolipoprotein Eb) detected was dramatically increased and peaked at the early intersexual stage. Another study has suggested that the expression of *apoeb* was significantly different between old and young female brains in zebrafish (*Danio rerio*) (Zhai et al., 2022). Furthermore, *SGK1* (integrin alpha 6) is responsible for regulating *foxo1* (forkhead box O1) (Ding et al., 2017), and *foxo1* knockdown suppressed expression of *sox9* (sex-determining region Y cox 9) (Kurakazu et al., 2019). Previous studies reported that *sox9* promoted testis cord formation during testicular differentiation (Li et al., 2014) and played an important role as a master regulator of Sertoli cell differentiation during testis development (Jakob and Lovel-Bafge, 2011). In addition, *sox9* was cloned in the lambari fish (*Astyanax altiparanae*) and evidenced its role in sex determination, sex differentiation, and the male reproductive cycle (Adolfi et al., 2015). Thus, we assume there is a complex regulatory mechanism of *sgk1* for male sex differentiation in *P. leopardus*.

Gonadal weight and gonadosomatic index are important indicators that reflect the degree of reproductive ability and gonadal development of fish and are useful to the mechanism research of reproductive biology and breeding. The GWASs on GW and GSI traits in this study showed that 22 candidate genes associated with sex-related traits were identified on chromosomes 5, 6, 10, 12, 13, 19, and 21. Moreover, sex as a covariate and unisex analysis were performed to avoid sex differences affecting the GW and GSI GWASs. It is noteworthy that several genomic loci and genes were identified simultaneously in different traits. All genes detected through the GWASs might play important roles in the gonadal development and differentiation of *P. leopardus. a2m* (alpha-2-macroglobulin) has been shown to be the regulatory gene in the development of the oviduct, which is stimulated in response to estrogen in chickens (*Gallus gallus*) (Lim et al., 2011; Lim and Song, 2014). *Usf1* (upstream stimulatory factor 1) is critical for the maintenance of germline stem cells in male mice (*Mus musculus*) (Faisal et al., 2019) and the control of gene expression during the onset of rat Sertoli cell differentiation (Wood et al., 2011). *hsd3b7* (3 beta-hydroxysteroid dehydrogenase type 7) was suggested to be involved in the estrogen-synthetic pathway in the zebra finch and considered primarily as a bile-synthesizing enzyme in mammals (London and Clayton, 2010). Furthermore, significant upregulation of *hsd3b7* in female clownfish (*Amphiprion clarkii*) provided obvious evidence for the importance of steroidogenesis in ovarian development and maintenance (Wang et al., 2022). *dll4* (delta-like protein 4) is a canonical Notch ligand in the Notch signaling pathway involved in mammalian sex determination and gonad development (Windley and Wilhelm, 2015; Whiteley et al., 2021). To investigate the effect of *xbp1* (x-box-binding protein 1) on steroidogenesis, knockdown of *xbp1* by RNAi was conducted in mouse granulosa cells and showed that *xbp1* depletion significantly affected the concentrations of estradiol (Wang et al., 2017). *tet3* (methylcytosine dioxygenase TET3) is involved in primordial germ-cell development, and interruption of the *tet3* gene could reduce germ cell numbers in zebrafish (Wang et al., 2021). Interestingly, primordial germ cells undergo significant DNA methylation reprogramming that is similar to the reprogramming during the adult zebrafish female-to-male sex transition (Wang et al., 2021). *esr1* (estrogen receptor alpha) is dispensable for sexual development and reproduction in medaka (*Oryzias latipes*) (Tohyama et al., 2017), and it can also modulate estrogen-induced sex reversal in the American alligator (*Alligator mississippiensis*) (Kohno et al., 2015). *gli3* (zinc finger protein GLI3) plays a key role in regulation of *dmrt3* (doublesex and Mab-3 related transcription factor 3), which was related to male sexual development in mice (*Mus musculus*) (Bellefroid et al., 2013). In addition, *gli3*, which resides at the intersection of hedgehog and androgen action, has been considered to be associated with male sex differentiation (Kothandapani et al., 2020). In our study, these associated genes were suggested to play an important role in sex differentiation and gonadal development.

## 5 Conclusion

Identification of reliable associated genomic and potentially interesting candidate genes affecting the sex differentiation and gonadal development process are important for parent selection and controlled breeding in *P. leopardus*. In the present GWAS analysis, multiple genomic loci and genes were identified as being significantly associated with the sex differentiation and gonadal development traits (including SDIF, GW, GSI, GW-CoSex, GSI-CoSex, FGW, MGW, FGSI, and MGSI) of *P. leopardus.* By comprehensively considering the associated peaks, GO annotation, KEGG pathway enrichment analyses, and biological information of genes, several candidate genes, such as *taf7*, *ddx6*, *apoeb*, *sgk1*, *a2m*, *usf1*, *hsd3b7*, *dll4*, *xbp1*, *tet3*, *esr1,* and *gli3*, provided insight into the investigation of not only the genetic mechanism of sex differentiation and gonadal development but also the sex control technologies in *P. leopardus*.

## Data Availability

The datasets presented in this study can be found in online repositories. The names of the repository/repositories and accession number(s) can be found in the article/Supplementary Material.
