# Supplementary material for: Genome-wide association study reveals genomic loci of sex differentiation and gonadal development in Plectropomus leopardus
Source: Front Genet. 2023 Aug 14;14:1229242. doi: 10.3389/fgene.2023.1229242 (PMC10461086; doi:10.3389/fgene.2023.1229242)
Supplement: Supplementary file 1 [file Presentation1.pdf]

Supplementary Material

Genome-wide association study reveals genomic loci of sex differentiation and gonadal development in Plectropomus leopardus

Jin Gao[1,2,3†], Yongbo Wang[1,2†], Jinye Liu[1,3], Fuxiao Chen[1,2], Yilan Guo[1], Hongji Ke[1], Xulei Wang[1], Ming Luo[1], Shuyuan Fu[1,2,3*]

*** Correspondence:** Shuyuan Fu: fushuyuan@hnhky.cn

1 Supplementary Figures and Tables

1.1 Supplementary Figures

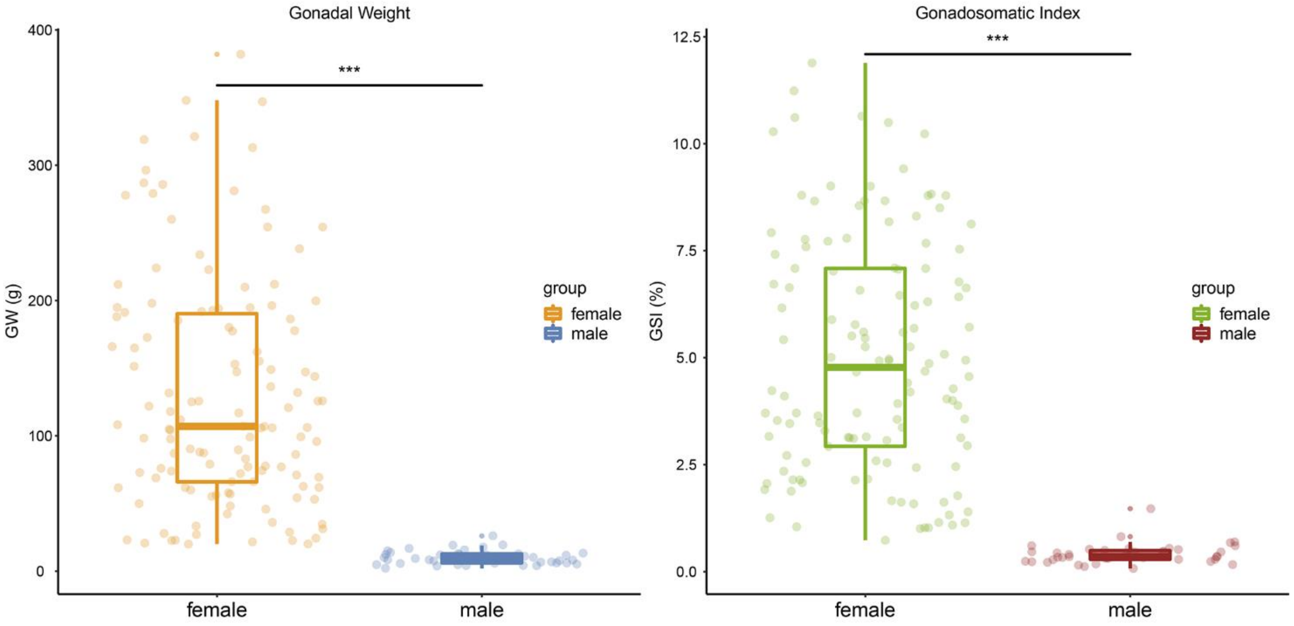

Supplementary Figure 1 | Boxplot showing comparison of the GW and GSI phenotypes for P. leopardus in female and male groups. *** indicates that there were significant differences ($p < 0.0001$) using the Wilcoxon test between the female and male groups of the GW and GSI phenotypes.

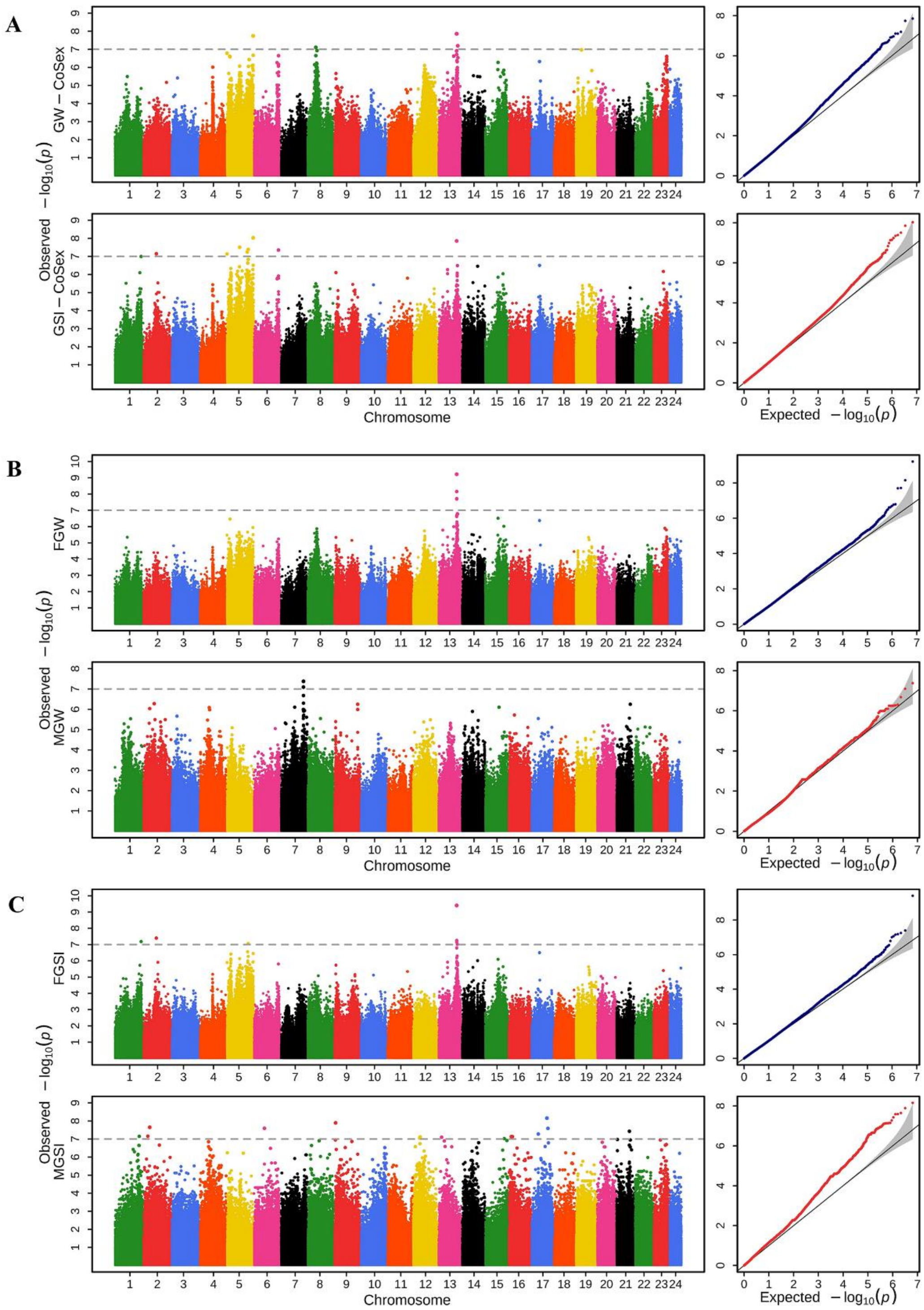

Supplementary Figure 2 | Manhattan and QQ plots of genome-wide SNP significance for the traits of (A) GW-CoSex and GSI-CoSex, (B) FGW and MGW, (C) FGSI and MGSI in *P. leopardus*.

1.2 Supplementary Tables

Supplementary Table 1. Genomic control values of each trait in GWAS.

Trait	Genomic Control Values
SDIF	1.04
GW	1.03
GSI	1.03
GW-CoSex	1.03
GSI-CoSex	1.04
FGW	1.04
MGW	0.98
FGSI	1.05
MGSI	1.27

Supplementary Table 2. The 46 SNPs of significant associated with sex differentiation and its related GWASs in *P. leopardus*.

Traits	Chr	SNP	Position (bp)	-log10(*P*)	Major Allele	Minor Allele
SDIF	1	SNP_1_27436225	27436225	7.37	T	G
	6	SNP_6_1266295	1266295	7.12	T	C
	7	SNP_7_41072598	41072598	7.37	C	T
	18	SNP_18_6022361	6022361	7.96	T	C
GW	6	SNP_6_35489335	35489335	8.14	G	C
	6	SNP_6_37599520	37599520	7.14	A	G
	6	SNP_6_37752485	37752485	7.72	A	T
	6	SNP_6_37752513	37752513	7.90	C	T
	6	SNP_6_37754971	37754971	7.59	T	C
	13	SNP_13_29949104	29949104	7.03	T	G
GW-CoSex	5	SNP_5_40241714	40241714	7.74	T	C
	8	SNP_8_12702498	12702498	7.11	G	C
	8	SNP_8_12702504	12702504	7.11	A	T
	13	SNP_13_28067114	28067114	7.86	C	G
	13	SNP_13_29728717	29728717	7.19	T	G
FGW	13	SNP_13_28067114	28067114	9.22	C	G
	13	SNP_13_28160201	28160201	7.70	T	C
	13	SNP_13_28160759	28160759	8.16	T	C
	13	SNP_13_28160799	28160799	7.72	T	C
MGW	7	SNP_7_34781521	34781521	7.09	T	C
	7	SNP_7_34796682	34796682	7.37	G	T
GSI	6	SNP_6_35489335	35489335	7.41	G	C
	6	SNP_6_37599520	37599520	7.66	A	G
	12	SNP_12_32393164	32393164	7.57	A	G
GSI-CoSex	2	SNP_2_19826396	19826396	7.15	T	C
	5	SNP_5_250437	250437	7.13	C	A
	5	SNP_5_19560616	19560616	7.51	A	G
	5	SNP_5_31248578	31248578	7.24	G	A
	5	SNP_5_32767410	32767410	7.39	C	G
	5	SNP_5_40241714	40241714	8.03	T	C
	6	SNP_6_37599520	37599520	7.35	A	G
	13	SNP_13_28067114	28067114	7.85	C	G

FGSI	1	SNP_1_40283653	40283653	7.18	C	T
	2	SNP_2_19826396	19826396	7.40	T	C
	5	SNP_5_32767357	32767357	7.06	T	C
	13	SNP_13_28067114	28067114	9.40	C	G
	13	SNP_13_28160759	28160759	7.15	T	C
	13	SNP_13_28160799	28160799	7.25	T	C
	13	SNP_13_29185517	29185517	7.01	C	T
MGSI	1	SNP_1_37383293	37383293	7.15	T	C
	2	SNP_2_6811953	6811953	7.15	A	T
	2	SNP_2_9738150	9738150	7.65	T	G
	6	SNP_6_16084515	16084515	7.59	T	C
	9	SNP_9_1891284	1891284	7.89	C	T
	12	SNP_12_10160189	10160189	7.05	C	G
	12	SNP_12_10774076	10774076	7.12	C	T
	12	SNP_12_10953531	10953531	7.12	T	A
	13	SNP_13_5010265	5010265	7.10	T	C
	15	SNP_15_29143275	29143275	7.02	C	G
	16	SNP_16_4010811	4010811	7.13	A	G
	16	SNP_16_5299965	5299965	7.13	G	T
	16	SNP_16_5783689	5783689	7.13	T	C
	17	SNP_17_10579604	10579604	7.28	C	T
	17	SNP_17_23824376	23824376	8.16	T	C
	17	SNP_17_25336837	25336837	7.59	A	G
	21	SNP_21_19871070	19871070	7.43	T	C

Supplementary Table 3. The annotation of each gene in TABLE 2.

Gene Name	Annotation
cops6	COP9 signalosome complex subunit 6
itga6	Integrin alpha-6
arhg6	Rho guanine nucleotide exchange factor 6
taf7	Transcription initiation factor TFIID subunit 7
apbp2	Amyloid protein-binding protein 2
brip1	BRCA1 interacting protein C-terminal helicase 1
ddx6	Probable ATP-dependent RNA helicase DDX6
apoeb	Apolipoprotein Eb
sgk1	Serine/threonine-protein kinase Sgk1
ppm1e	Protein phosphatase 1E
a2m	Alpha-2-macroglobulin
usf1	Upstream stimulatory factor 1
mtnr1b	Melatonin receptor type 1B (Fragment)
akap1	A-kinase anchor protein 1, mitochondrial
hsd3b7	3 beta-hydroxysteroid dehydrogenase type 7
dll4	Delta-like protein 4
lce	Low choriolytic enzyme
xbp1	X-box-binding protein 1
tet3	Methylcytosine dioxygenase TET3
npffr2	Neuropeptide FF receptor 2
exoc8	Exocyst complex component 8
arv1	Protein ARV1

sprtn	SprT-like domain-containing protein Spartan
cetp	Cholesteryl ester transfer protein
mkb	Midkine-B
sall1	Sal-like protein 1
cnot3	CCR4-NOT transcription complex subunit 3
sphk2	Sphingosine kinase 2
esr1	Estrogen receptor alpha
oprk1	Kappa-type opioid receptor
gli3	Zinc finger protein GLI3
